# Extracellular vesicle miRNAs in breast milk of obese mothers

**DOI:** 10.3389/fnut.2022.976886

**Published:** 2022-10-12

**Authors:** Young Eun Cho, Rany Vorn, Michael Chimenti, Keith Crouch, Chen Shaoshuai, Janhavi Narayanaswamy, Alaria Harken, Reegan Schmidt, Jessica Gill, Hyangkyu Lee

**Affiliations:** ^1^College of Nursing, The University of Iowa, Iowa City, IA, United States; ^2^School of Nursing, Johns Hopkins University, Baltimore, MD, United States; ^3^College of Medicine The University of Iowa, Iowa City, IA, United States; ^4^Department of Neurology and Neurosurgery, The Johns Hopkins University School of Medicine, Baltimore, MD, United States; ^5^Mo-Im Kim Nursing Research Institute, College of Nursing, Yonsei University, Seoul, South Korea

**Keywords:** breast milk, maternal obesity, extracellular vesicles, exosomes, miRNAs

## Abstract

**Background:**

Breast milk has abundant extracellular vesicles (EVs) containing various biological molecules (cargo), including miRNAs. EVs are not degraded in the gastrointestinal system and circulation; thus, breast milk EVs (bEVs) are expected to interact with other organs in breastfed infants and modify the gene expression of recipient cells using miRNAs. Maternal pre-pregnancy BMI is a critical factor influencing the composition of breast milk. Thus, in mothers with obesity, miRNAs in bEVs can be altered, which might be associated with adverse health outcomes in infants. In this study, we examined 798 miRNAs to determine which miRNAs are altered in the bEVs of mothers with obesity and their potential impact on breastfed infants.

**Methods:**

We recruited healthy nursing mothers who were either of normal weight (BMI < 25) or with obesity (BMI ≥ 30) based on their pre-pregnancy BMI, and delivered a singleton baby in the prior 6 months. EVs were isolated from breast milk with ultracentrifugation. bEV characteristics were examined by flow cytometry and fluorescence imaging of EV markers. A total of 798 miRNAs were screened using a NanoString human miRNA panel to find differentially expressed miRNAs in bEVs of mothers with obesity compared to mothers of normal weight.

**Results:**

We included 65 nursing mothers: 47 of normal weight and 18 with obesity based on pre-pregnancy BMI. After bEV isolation, we confirmed the expression of various EV markers. Out of 37 EV markers, CD326 (EpCaM) was the most highly expressed in bEVs. The most abundant miRNAs in bEVs include *miR-30b-5p, miR-4454, miR-494-3p*, and *let-7 miRNAs*. Target genes of the top 10 miRNAs were associated with cancer, prolactin pathway, EGFR, ErbB, and FoxO signaling pathway. In bEVs of mothers with obesity, 19 miRNAs were differentially expressed (adjusted *p* < 0.05 cut-off), which include *miR-575, miR-630, miR-642a-3p*, and *miR-652-5p*. These miRNAs and their target genes were associated with neurological diseases and psychological disorders.

**Conclusion:**

In this study, we characterized bEVs and demonstrated altered miRNAs in bEVs of mothers with obesity and identified the pathways of their potential target genes. Our findings will provide insight for future studies investigating the role of bEVs in breastfed infants.

## Introduction

Extracellular vesicles (EVs) are nanosized particles (30–150 nm in diameter) released from cells (1). EVs encapsulate biological molecules (cargos) of donor cells during the biogenesis of EVs, which include non-coding RNAs, lipids, and proteins. Because EVs are surrounded by a lipid bilayer, they are not degraded in the gastrointestinal system and circulation. Thus, EVs can interact with other cells and modify the gene expression of recipient cells by transferring internal cargo molecules such as miRNAs (2). Milk of mammals has abundant EVs (3, 4). After oral administration, milk EVs are distributed in the intestine and other distal organs, including the liver, spleen, heart, and brain (4, 5). Thus, human milk EVs and EV cargos are considered to interact with multiple organs and play a significant role in the growth and development of breastfed infants. Furthermore, milk EVs from humans and other mammals are emerging as therapeutics (6, 7). In several pathological conditions such as ulcerative enterocolitis and cancers, it has been demonstrated that milk EVs enhance immunity and suppress inflammation (8, 9). The therapeutic characteristic of milk EVs may be associated with the miRNA profiles found in milk EVs (10). However, the signaling mechanism associated with miRNAs of milk EVs and the major role players among miRNAs in EVs have not been fully elucidated. Therefore, understanding breast milk EVs (bEVs) and their miRNA profiles is critical not only to increasing our knowledge about bEV dynamics in infants but also to developing new therapeutics using bEVs.

The population of women with obesity of childbearing age has significantly increased in the US (11, 12). Increased maternal BMI critically influences the component of breast milk. In mothers with overweight and obesity, lipids, metabolites, and immunological components of breast milk were dysregulated, which are associated with adverse impacts on infants’ physical/mental health and development (13–15). miRNAs are also altered in mothers with overweight and obesity. Especially altered miRNAs encapsulated by EVs can cross biological barriers and be internalized into other distal organs without degradation (10, 16). Thus, it is critical to understand which miRNAs are altered in mothers with obesity, and their potential impact on breastfed infants. Recently, a few selected miRNAs associated with adipogenesis and glucose metabolism were tested in bEVs from mothers with overweight and obesity (17). *miR-148a* and *miR-30b* were downregulated in mothers with overweight and obesity, which are related to the body composition of infants. However, this study examined only a few miRNAs, which could not provide a broader view of miRNAs in bEVs that are altered in maternal obesity and their potential impacts. In this study, we compared the expression level of 798 biologically relevant miRNAs in bEV between mothers with obesity and mothers of normal weight and analyzed enriched pathways of differentially expressed miRNAs found in mothers with obesity. Our findings provide a comprehensive understanding of the altered bEV miRNAs in maternal obesity and their potential impacts on breastfed infants.

## Materials and methods

### Participants

We recruited healthy nursing mothers who were ≥18 years old and had delivered a full-term (37–40 weeks) singleton newborn within the prior 6 months. Mothers whose pre-pregnancy BMI was either <25 (normal weight) or ≥30 (obesity) were included in this analysis. Mothers with chronic conditions that might affect body weight changes, such as chronic diabetes or thyroid diseases, were excluded. After electronic informed consent was obtained, a breast milk sample collection kit was mailed to the participant’s home with detailed instructions. After 1 h of fasting, participants collected 50 ml of breast milk and froze it in their home freezer. Within 48 h of sample collection, samples were returned to the lab and stored at −80°C until use. Participants also completed an online demographic survey and the Automated Self-Administered 24-hour (ASA24) Dietary Assessment Tool (18). All procedures were followed under an approved IRB protocol (IRB #202005237).

### Breast milk extracellular vesicle isolation and characterization

Breast milk EVs were isolated using ultracentrifugation. First, frozen breast milk was thawed at 4°C overnight. Breast milk (15 ml) was centrifuged at 3,000 *g* × 3 to remove bulk fat. The remaining supernatant was then centrifuged at 12,000 *g* for 40 min to remove the remaining fat and cell debris. After filtering with a 0.22-μm syringe filter, samples were centrifuged at 100,000 *g* for 2 h to pellet EVs. The EV pellet was resuspended in 300 μl sterile PBS and rotated overnight at 4°C. EV characteristics were tested using the ExoView R100 platform (NanoView Bioscience, MA, United States), which allowed us to measure the expression of general EV markers, including CD81, CD63, and CD9, using fluorescence antibodies. The number of particles labeled by fluorescence antibodies was counted, and their size was measured. In addition, we tested surface epitopes of bEVs using the MACSPlex Exosome Kit (Miltenyi Biotec, Germany), which can detect up to 37 surface epitopes of EVs. Flow cytometric analysis was performed with a MACSQuant Analyzer 10 flow cytometer equipped with 405, 488, and 638-nm lasers and a built-in 96-well plate reader (Miltenyi Biotec, Germany).

The expression level of CD326 and TSG101 was examined using a western blot. The protein level of EVs was measured using a Qubit protein assay kit (ThermoFisher Scientific, MA, United States), and equal amounts of protein were loaded on SDS-PAGE gel. Proteins were transferred to a PVDF membrane and blocked with a 5% skim milk solution for 1 h. The membrane was then incubated with a CD326 antibody (Santa Cruz Biotechnology, CA, United States) and TSG101 antibody (ThermoFisher Scientific, MA, United States) overnight at 4°C. Signals were detected with an HRP-conjugated antibody (Santa Cruz Biotechnology, CA, United States) using the ECL-Pico system (ThermoFisher Scientific, MA, United States).

### miRNA profiling with NanoString miRNA assay

After NanoString assay was performed, data were obtained as “RCC” format files. These were imported into R with the *readRcc* function from the NanoString QCPro package (19). We followed the “Remove-Unwanted-Variation-genes” (RUVg) normalization procedure for NSolver data outlined in Love et al. (20). RUVg normalization uses negative control genes (RUVg) to remove unwanted technical and batch variation in the data, and has been shown to be superior to the NSolver-provided normalization procedure (21). All samples passed nSolver QC checks (“imaging,” “binding density,” “linearity of positive controls,” and “Limit of detection”) and were included in the analysis. Next, the five housekeeping genes on the panel were assessed for correlation with sample BMI; no statistically significant correlation was detected for any of the five genes. We also check the technical batches for the number of genes below the limit of detection (LOD). Batch 4 (of 9) did show a higher proportion of endogenous genes below the LOD (data not shown), but we chose to retain the samples in the analysis and remove the unwanted variation through the normalization procedure.

Remove-Unwanted-Variation-genes normalization was applied to the raw NSolver data. Normalized data were visualized with relative log expression (RLE) and principal component analysis (PCA) plots for different values of “k” (*k* = 1,2,3). RLE and PCA plots on RUVg normalized data showed clear removal of systematic technical effects owing to batch in the data, particularly at *k* = 2 and *k* = 3 (data not shown). Thus, RUVg-normalized data with *k* = 2 was chosen for downstream differential expression analysis. A DESeq2 object was created from the raw data using *DESeqDataSetFromMatrix* with design conditioned on the BMI factor variable, the postpartum date quartile, and the RUVg (*k* = 2) “W1” and “W2” correction factors learned for technical batch effects. DE analysis proceeded according to best practices as described in the DESeq2 vignette. The R scripts used for exploratory analysis and final DE analysis are available on Github (https://github.com/mchimenti/project_nanostring_miRNA_ breastmilk_jan2022/blob/115a6577559186a02b466d3cff1fa38d8258c782/nanostring_deseq2_analysis_cho_jan2022.Rmd and https://github.com/mchimenti/project_nanostring_miRNA_ breastmilk_jan2022/blob/115a6577559186a02b466d3cff1fa38d8258c782/nanostring_deseq2_analysis_Version2_cho_Mar2022.Rmd, respectively).

### Statistical analysis

Demographics and the expression of EV markers between groups were compared by the Mann-Whitney U test with a statistical threshold of *p* = 0.05 using SPSS 27 (IBM, NY, United States). Ingenuity Pathway Analysis (Qiagen, Germany) was used to analyze network interactions among differentially expressed miRNAs and their target genes. KEGG pathway enrichment analysis was performed using DAVID online tools^1^ (22, 23). Using a Benjamini–Hochberg method, the false discovery rate of the pathway analysis was calculated in the KEGG enrichment pathway analysis. All plots were generated using GraphPad Prism 7 (GraphPad Software, Inc., CA, United States).

## Results

We analyzed 65 human breast milk samples from healthy nursing mothers who had delivered a baby within the prior 6 months. The mean age of participants was 32.7 years, and 92% of the participants were White. We included mothers whose pre-pregnancy BMI was <25 (normal weight, *n* = 47) and ≥30 (obesity, *n* = 18). Breast milk samples were collected an average of 66 days postpartum. Detailed demographic characteristics are described in Table 1, and metadata is in the Supplementary Document.

**TABLE 1:** Subject demographics.

	All participants	Lean (pre-pregnancy BMI < 25, *n* = 47)	Obese (pre-pregnancy BMI≥ 30, *n* = 18)	*p* value
**Age (years)**	32.7 ± 3.7	33.0 ± 3.8	32.1 ± 3.4	0.396
**Race**				
White	60 (92.3%)	47 (91.5%)	18 (94.4%)	0.385
Asian	2 (3.1%)	2 (4.3%)	0	
African American	1 (1.5%)	0	1 (5.6%)	
Multiracial	1 (1.5%)	1 (2.1%)	0	
Unknown	1 (1.5%)	1 (2.1%)	0	
**Pre-pregnancy BMI**	25.2 ± 5.6	22.0 ± 1.9	33.7 ± 2.5	<0.001
**Current BMI**	26.8 ± 5.1	24.1 ± 2.8	33.8 ± 2.4	<0.001
**Body weight changes during pregnancy (Ibs)**	29.1 ± 9.5	31.8 ± 8.4	22.2 ± 9.1	<0.001
**Postpartum sample collection (days)**	65.9 ± 49.0	65.9 ± 46.0	65.8 ± 57.6	0.363
**Parity**				
1	14 (21.2%)	10 (21.7%)	4 (22.2%)	0.555
2	30 (45.5%)	23 (50.0%)	7 (38.9%)	
3	16 (24.2%)	11 (23.9%)	5 (27.8%)	
4 or more	4 (6.2%)	2 (4.3%)	2 (11.1%)	
**Gestation period (weeks)**	39.1 ± 1.2	39.0 ± 1.3	39.1 ± 0.8	0.606
**Delivery mode**				
C-section	9 (13.8%)	6 (12.8%)	3 (16.7%)	0.767
Vaginal delivery	56 (86.2%)	41 (87.2%)	15 (83.3%)	
**Nursing methods**				
Direct breastfeeding	57 (87.7%)	43 (91.5%)	14 (77.8%)	0.132
Bottled feeding	35 (53.8%)	22 (46.8%)	13 (72.2%)	0.066
Formula feeding	10 (15.4%)	6 (12.8%)	4 (22.2%)	0.344
**Pregnancy complication**				
Pre-eclampsia	7.7% (*n* = 5)	1 (2.2%)	4 (22.2%)	0.007
Gestational diabetes	4.6% (*n* = 3)	1 (2.2%)	2 (11.1%)	0.128
**Average intake per day (cal)**	2,438.5 ± 773.4	2435.7 ± 760.9	2446.1 ± 830.4	0.964

**FIGURE 1:**
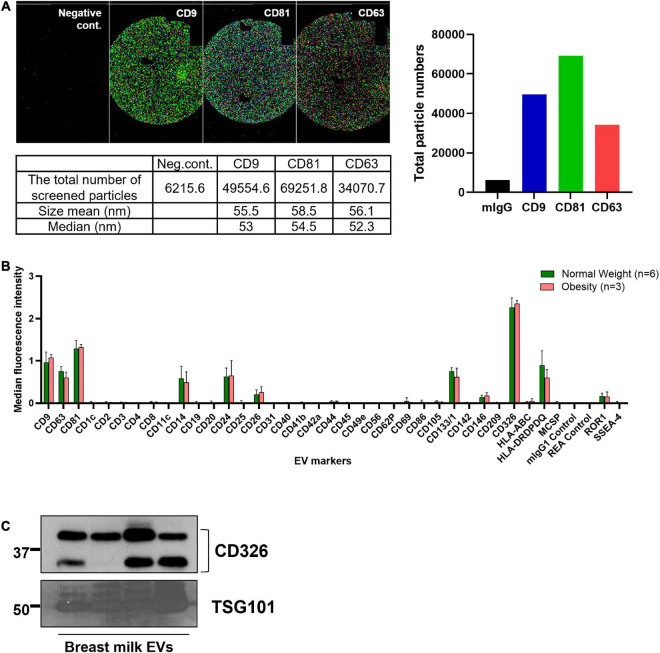
Characteristics of breast milk EVs (bEVs). **(A)** Particles expressing three EV markers (CD9, CD81, and CD63) were examined using NanoView R100 (*n* = 8). The representative images show the colocalization of these three EV markers; CD9 in dark blue; CD81 in green; and CD63 in red. mIgG is a negative control. The size range of EVs expressing these markers is 50–60 nm. **(B)** The median fluorescence intensity of EV markers was calculated after background correction with negative controls, and the intensity was compared between groups. **(C)** The expression level of CD326 and TSG101 in bEVs was confirmed with western blotting (*n* = 4).

The presence of EVs was confirmed by the expression of EV markers, including CD9, CD63, CD81, and TSG101, and by the size of the particles (Figure 1A). According to the analysis of surface epitopes, CD14, CD24, CD133/1, CD326, and HLA-DRDPDQ were highly expressed in bEVs, in addition to the general EV markers CD9, CD63, and CD81. Out of 37 markers analyzed, CD326 was the most abundant EV marker expressed in bEVs. The expression level of CD326 was also confirmed with western blotting (Figures 1B,C).

**FIGURE 2:**
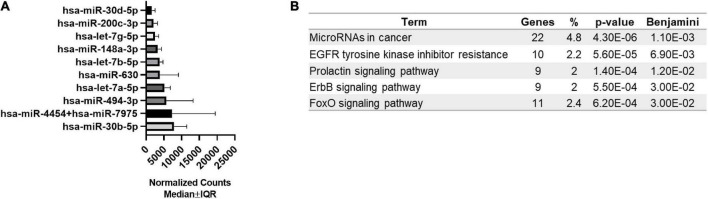
Top 10 most abundant miRNAs in bEVs and enriched pathways of target genes. **(A)** The top 10 most abundant miRNAs were identified using normalized median counts (*n* = 65). **(B)** Enriched pathways of target genes of the top 10 miRNAs of bEVs are demonstrated.

Next, we examined the expression level of 798 miRNAs and analyzed the most abundant miRNAs in bEVs. All 798 miRNAs were detected and included in the differentially expressed miRNA analysis. The top 10 most abundant miRNAs are listed in Figure 2, including *miR-30b-5p, miR-4454* + *miR-7975, miR-494-3p*, and *let-7a-5p*. Enrichment pathways analysis with target genes of top 10 bEV miRNAs demonstrated that cancer, epidermal growth factor receptor (EGFR), prolactin signaling pathway, erythroblastic leukemia viral oncogene homologue (ErbB), and forkhead box, class O (FoxO) signaling pathways are enriched (Figure 2B). The top 10 most abundant bEV miRNAs were the same between groups with normal weight and obesity, except for one miRNA (Supplementary Figure 1). The expression level of the top 10 most abundant miRNAs was compared between groups, demonstrating that there was no significant difference (Supplementary Figure 2).

**FIGURE 3:**
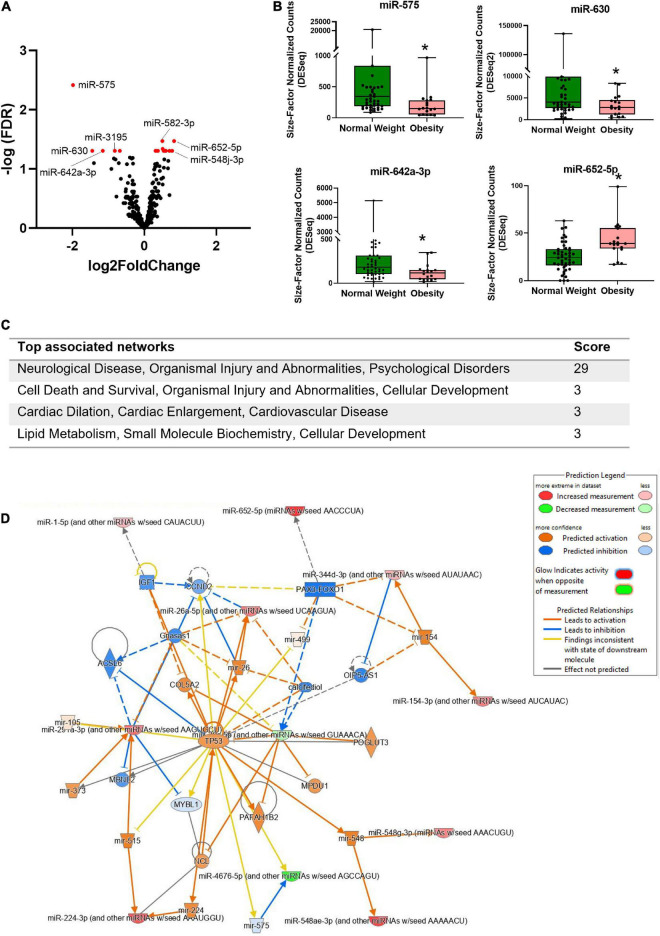
Differentially expressed miRNAs in bEVs of mothers with obesity. **(A)** Volcano plot shows the distribution of miRNAs in bEVs with fold changes and *p* values. miRNAs in red represent deregulated miRNAs with a cut-off of adjusted *p* < 0.05. **(B)** Normalized counts of selected miRNAs are shown between groups. *Represents a statistical significance with adjusted *p* values < 0.05. **(C)** Top associated networks are analyzed using differentially expressed miRNAs and their predicted target genes in bEVs of mothers with obesity. **(D)** The network is the #1 network in panel **(C)**; neurological disease, organismal injury and abnormalities, and psychological disorders. TP53 is a hub gene of this network.

Differentially expressed miRNAs in bEVs of mothers with obesity were analyzed after controlling for postpartum dates when samples were collected. Nineteen miRNAs were significantly differentially expressed in bEVs of mothers with obesity, including *miR-575*, *miR-630, miR-642a-3p*, and *miR-652-5p* (Figure 3A, B). The median and interquartile range (IQR) of the normalized counts of these miRNAs are shown in Table 2. The top network of these miRNAs and their predicted target genes was associated with neurological diseases and psychological disorders (Figure 3C). In this network, the hub gene was TP53, which is predicted to be activated (Figure 3D).

**TABLE 2:** Differentially expressed miRNAs in breast milk EVs of mothers with obesity (pre-pregnancy BMI ≥ 30) compared to mothers with normal weight (pre-pregnancy BMI < 25).

	Normal weight (*n* = 47)		Obesity (*n* = 18)		Adjusted *p* value	log2Fold change
	Median	IQR	Median	IQR		
*miR-575*	345	646	150.5	224	0.004	−1.983
*miR-630*	4,038	7236	2796.5	3074	0.049	−1.447
*miR-642a-3p*	182	211	115.5	103	0.049	−1.154
*miR-652-5p*	24	17	39	21	0.034	0.825
*miR-3195*	16	13	11.5	6	0.049	−0.82
*miR-548j-3p*	1,146	1187	2159	1719	0.049	0.77
*miR-522-3p*	449	351	634	444	0.049	0.696
*miR-30c-5p*	161	122	116	43	0.049	−0.679
*miR-448*	215	137	65	55	0.049	0.595
*miR-302b-3p*	292	181	451	204	0.049	0.593
*miR-219b-3p*	130	62	169	86	0.049	0.579
*miR-1297*	569	356	775.5	392	0.049	0.556
*miR-487a-3p*	73	47	115.5	70	0.049	0.544
*miR-499b-3p*	67	39	105	59	0.049	0.539
*miR-548g-3p*	308	145	403.5	148	0.046	0.509
*miR-582-3p*	47	25	71	26	0.034	0.502
*miR-450b-5p*	56	19	66.5	24	0.049	0.378
*miR-410-3p*	25	7	29.5	12	0.049	0.325
*miR-1-5p*	56	18	71	15	0.049	0.312

## Discussion

miRNAs of bEVs are considered to play an important role in the growth and development of infants. Maternal BMI influences miRNA profiles; thus, knowing about altered miRNAs of bEVs in maternal obesity is critical for better understanding the role of breast milk in infants (10). Furthermore, it has recently been suggested that bEVs have therapeutic properties for multiple pathological conditions, including intestinal diseases and cancers (7). Because major EV cargos are miRNAs, miRNAs are considered major role players in the therapeutic effect. Thus, profiling miRNAs in bEVs will help figure out the potential signaling pathways of therapeutic effect.

Mammary epithelial cells are thought to be the most dominant cells contributing to breast milk production (24, 25). We found that bEVs expressing CD326 (EpCAM) are the most abundant in breast milk. CD326 is an epithelial cell adhesion molecule used as a marker for epithelial cells in body fluids (26). The ectodomain of CD326, EpEX, is a ligand of EGFR, which is highly expressed in the mammalian intestinal epithelium (27, 28). EGFR is a key signaling molecule involved in intestinal cell growth, repair, and migration (29). Although non-specific EV uptake is generally shared in most cell types, CD326 expression on bEVs might enhance the interaction between bEVs and intestinal epithelial cells of infants. In addition, it was recently revealed that CD326 regulates intestinal epithelial integrity and stem cells. CD326 enhanced the growth of intestinal epithelial cell organoids and spheroids (30). Multiple studies have demonstrated that milk EVs improve pathological intestinal conditions (7, 8, 31), and miRNAs are expected to play a critical role in the therapeutic impact of milk EVs. However, considering the high expression of CD326 and the role of CD326 in the intestine, the contribution of CD326 to the therapeutic impact of milk EVs may need to be examined further.

In order to understand the content of bEVs, several studies have profiled miRNAs of bEVs. There are differences across the studies in the characteristics of mothers, breast milk collection time, EV isolation methods, and miRNA profiling platforms, therefore, the results of studies are inconsistent. Despite that, many studies have shown that *miR-148a-3p, let-7 miRNAs*, and *miR-200c-3p* are abundantly expressed in bEVs, which are associated with promoting immune function and inflammatory regulation (32–35). Meanwhile, another study shows completely different abundant miRNAs, such as *miR-4271, miR-3197*, and *miR-2861* (36). These are involved in the estrogen signaling pathway, ECM-receptor pathway, axon guidance, and various cancers. Our study found that *miR-200c-3p, miR-148a-3p*, and multiple *let-7 miRNAs* including *let-7a-5p, let-7b-5p, and let-7g-5p* are abundant in bEVs as many other studies have demonstrated. However, we also found different miRNAs, for example, *miR-4454* + *miR-7975*. These two miRNAs were grouped together in this platform because the mature sequence of *miR-7975* differs by only one base from *miR-4454* (37). Previous reports showed that *miR-4454* promotes inflammation or cancer invasion and migration (38, 39). However, the role of either *miR-4454* or *miR-7975* in breast milk for infants’ health and development is relatively unknown. One study reported that *miR-4454* was deregulated in mothers of premature infants compared to mothers of term infants, however, the role of this miRNA was not elucidated (40). In order to fully understand the content of bEVs and the role of bEVs on infants’ health, more studies with controlled sample collection methods and profiling platforms would be needed.

The top 10 most abundant miRNAs identified in bEVs are associated with cancers and prolactin signaling pathways, which might be related to mammary epithelial cells. As shown in Figure 1, the majority of bEVs originated from mammary epithelial cells. Most human breast cancers arise from the epithelial cells (41), and prolactin stimulates mammary epithelial cells to synthesize milk components (42). Besides that, EGFR, ErbB, and FoxO signaling pathways were identified as enriched patyways in miRNAs of bEVs. EGFR and ErbB2 signaling lead to downstream activation of FoxO3, which regulates gene expression affecting apoptosis, cell-cycle control, glucose metabolism, and oxidative stress (43). Activation of these molecules is involved in various disease mechanisms, including cancers, type 2 diabetes, and cardiovascular diseases (44, 45). Abundant miRNAs in bEVs might inhibit the role of their target genes in the recipient cells, and resulting in a beneficial effect (46). In fact, previous studies demonstrated that abundant miRNAs in bEVs, including *miR-30b-5p, miR-494-3p*, *miR-148a-3p*, and the *let-7 miRNAs*, suppress tumor progression and development and reduce inflammation (47–49). In intestinal epithelial cells, bEVs inhibited the TLR4/NFκB and NLR4 inflammasome pathways and reduced inflammation (8). EGFR and TLR4 pathways are interrelated and target transcription factors such as NFκB (50, 51). Further studies to define the precise downstream pathways modulated by bEVs will deepen our understanding of the beneficial role of bEVs.

Previously, studies have shown altered breast milk miRNAs in increased maternal pre-pregnancy BMI (36). The expression patterns of miRNAs related to leptin and adiponectin, including *miR-222*, *miR-103*, *miR-17*, *miR-let-7a*, and *miR-let-7c*, were different in mothers with overweight and obesity compared to those in mothers with normal weight (52). In bEVs of mothers with overweight and obesity, *miR-148a* and *miR-30b* related to adipogenesis and glucose metabolism, were downregulated compared to mothers with normal weight (17). Interestingly, the expression level of these miRNAs was correlated with infants’ weight, fat mass, and body fat (%), thus, the authors suggested that these miRNAs might protect infants from obesity and type 2 diabetes later in life. In our study, these miRNAs are abundantly expressed in bEVs, however, their expression levels were not significantly different between groups. Different EV isolation methods, different RNA detection platforms, and different postpartum dates when breast milk was collected might influence this discrepancy. We identified several other differentially expressed miRNAs in mothers with obesity, associated with adiposity and obesity, such as *miR-642a*, *miR-30c*, *miR-448*, and *miR-302b* (53, 54). These miRNAs might play a role in either disturbing the metabolism of infants or protecting infants from harmful outcomes.

However, most previous studies that examined altered breast milk components in mothers with obesity demonstrated different results. It is reported that altered fatty acids in mothers with obesity have decreased neuroprotective factors and are associated with the cognition of infants (14, 55). Altered metabolites of breast milk in mothers with overweight and obesity were related to neurodegenerative diseases and neuropsychiatric disorders (56). We also found that altered miRNAs of bEVs in mothers with obesity are involved in neurological and psychological disorders. Interestingly, TP53 was a hub gene of this network and was predicted to be activated. TP53 encodes p53, a well-known cancer suppressor, which plays a critical role in brain development and neural stem cell regulation (57). Previous reports demonstrated that altered regulation of TP53 can lead to various neurological/psychological disorders such as Alzheimer’s disease, schizophrenia, and encephalopathy (58–60). Our findings substantiate these previous reports about the potential impact of altered breast milk components in mothers with obesity on the neurological development of breastfed infants (17). A pre-clinical longitudinal study should be performed to elucidate the long-term health outcomes in infants associated with altered miRNA in bEVs of mothers with obesity.

bEVs also provide information about the breasts of nursing mothers. Most bEVs originate from mammary epithelial cells, where most breast cancer arises. Especially, women with obesity are at increased risk of breast cancers, therefore, differentially expressed miRNAs of bEVs in mothers with obesity could be the molecules that either help us to predict breast cancer occurrence or trigger cancer development. In fact, previous studies demonstrated that downregulated miRNAs in bEV of mothers with obesity, such as *miR-630* and *miR-642*, suppress cancers (61, 62). *miR-548j-3p*, upregulated in mothers with obesity, plays a role as a metastasis promoter in breast cancer (63). However, some other miRNAs known as tumor suppressors, including *miR-548-3p* and *miR-652-5p* (64, 65), were slightly upregulated in mothers with obesity. Further studies would be needed to test each miRNA as a breast cancer predictive biomarker for mothers with obesity. More importantly, these miRNAs are eventually transferred to breastfed infants. The impact of these altered cancer-related miRNAs on infants also needs to be considered.

Our study has limitations. First, although it is a pilot study, we haven’t been able to include enough samples in the group with obesity. However, we excluded the effect of the overweight population, which may diminish the significance of the result. Second, we analyzed breast milk collected from a wide range of postpartum dates. However, we used mature breast milk that was relatively stable in composition (66), and we statistically controlled for the potential impact of different postpartum phases. Third, there is a variation in time between milk collection and the analysis of bEV miRNA. While miRNAs are globally stable, individual miRNAs display rapid decay dynamics in some specific situations (67). Although we froze breast milk at –80°C until use and did not repeat the freeze and thaw cycle after RNA extraction until NanoString analysis, there might be a possibility of miRNA degradation that can affect the miRNA profiles.

To conclude, we characterized bEV miRNAs and identified differentially expressed miRNAs of bEVs in mothers with obesity and demonstrated altered miRNAs in bEVs. Altered miRNAs in bEVs of mothers with obesity may influence the neurological and psychological development of breastfed infants. Further longitudinal studies investigating the precise role of altered miRNAs in infants need to be followed.

## Data availability statement

The datasets presented in this study can be found in online repositories. The names of the repository/repositories and accession number(s) can be found below: https://www.ncbi.nlm.nih.gov/geo, GSE212951.

## Ethics statement

The studies involving human participants were reviewed and approved by the Institutional Review Board of the University of Iowa (IRB #202005237). The patients/participants provided their written informed consent to participate in this study.

## Author contributions

YEC: conceptualization, methodology, resources, writing—original draft preparation, supervision, and funding acquisition. YEC and MC: formal analysis and data curation. YEC, RV, KC, CS, JN, AH, and RS: investigation. YEC, RV, CS, JG, and HL: writing—review and editing. YEC and RV: Visualization and supervision. All authors contributed to the article and approved the submitted version.

## Footnotes

1
https://david.ncifcrf.gov/
