# Supplementary material for: Extracellular vesicle miRNAs in breast milk of obese mothers
Source: Front Nutr. 2022 Oct 12;9:976886. doi: 10.3389/fnut.2022.976886 (PMC9597365; doi:10.3389/fnut.2022.976886)
Supplement: Supplementary file 2 [file Data_Sheet_1.docx]

**Supplementary information**

**Supplementary Table 1. Deregulated miRNAs in breast milk EVs of obese mothers (pre-pregnancy BMI ≥ 30) compared to lean mothers (pre-pregnancy BMI < 25)**

|  | **Adjusted *p* value** | **log2 Fold Change** |
| --- | --- | --- |
| hsa-miR-575 | 0.004 | -1.983 |
| hsa-miR-652-5p | 0.034 | 0.825 |
| hsa-miR-582-3p | 0.034 | 0.502 |
| hsa-miR-548g-3p | 0.046 | 0.509 |
| hsa-miR-1297 | 0.049 | 0.556 |
| hsa-miR-302b-3p | 0.049 | 0.593 |
| hsa-miR-30c-5p | 0.049 | -0.679 |
| hsa-miR-499b-3p | 0.049 | 0.539 |
| hsa-miR-548j-3p | 0.049 | 0.77 |
| hsa-miR-3195 | 0.049 | -0.82 |
| hsa-miR-450b-5p | 0.049 | 0.378 |
| hsa-miR-448 | 0.049 | 0.595 |
| hsa-miR-522-3p | 0.049 | 0.696 |
| hsa-miR-487a-3p | 0.049 | 0.544 |
| hsa-miR-410-3p | 0.049 | 0.325 |
| hsa-miR-642a-3p | 0.049 | -1.154 |
| hsa-miR-630 | 0.049 | -1.447 |
| hsa-miR-219b-3p | 0.049 | 0.579 |
| hsa-miR-1-5p | 0.049 | 0.312 |

**A. B.**
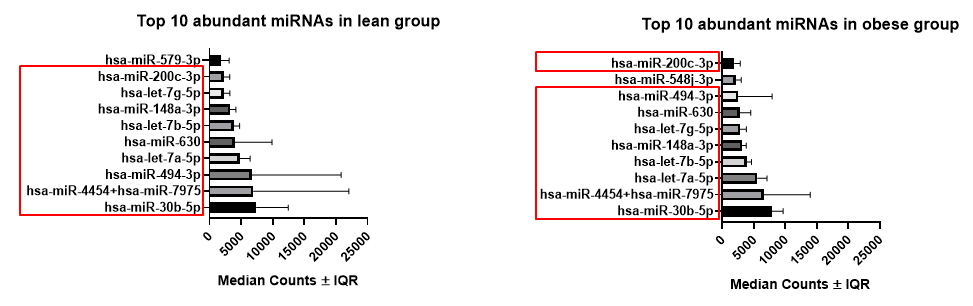


**Supplementary Figure 1.** **Median of normalized counts of the top 10 most abundant miRNAs in lean group (A, n=47) and obese group (B, n=18).** miRNAs in a red box are common miRNAs between groups.


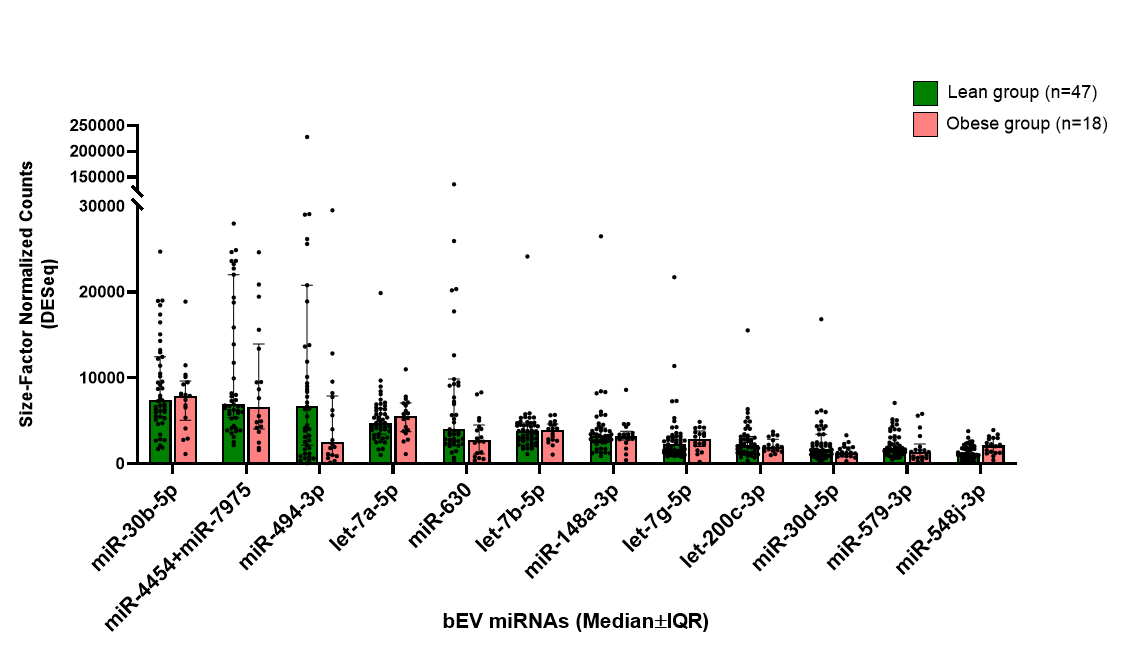


**Supplementary Figure 2.** **Comparison of the most abundant miRNAs between the lean group (n=47) and the obese group (n=18).**
